# Interesting features of Cardiac Magnetic Resonance in a Case of Hypereosinophilic Syndrome Secondary to Toxocariasis

**DOI:** 10.1002/ccr3.5043

**Published:** 2021-11-07

**Authors:** Sanaz Asadian, Nahid Rezaeian, Sahar Asl Fallah

**Affiliations:** ^1^ Rajaie Cardiovascular Medical and Research Center Iran University of Medical Sciences Tehran Iran

**Keywords:** Cardiac Magnetic Resonance, hypereosinophilia, Toxocariasis

## Abstract

Toxocariasis is a relatively common parasitic disease that can rarely affect the heart. Cardiac toxocariasis may lead to restrictive cardiomyopathy secondary to hypereosinophilia. CMR is a valuable diagnostic method in hypereosinophilic syndrome.

## Graphical abstract

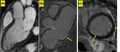

## 1 INTRODUCTION

We describe a 49‐year‐old man with established systemic toxocariasis, for whom serology results and cardiac magnetic resonance (CMR) imaging findings confirmed endomyocardial involvement in the context of toxocariasis‐induced hypereosinophilia. We recommend CMR with a lower threshold for the prompt detection and treatment of possible cardiac complications.

Toxocariasis is a known parasitic disease caused by *Toxocara canis* and *Toxocara cati*. The most common clinical findings are constitutional and allergic symptoms.^1^ On laboratory data, hypereosinophilia (HE) is almost always present in infected patients. Cardiac involvement has been reported in patients with toxocariasis, with myocarditis being the most common presentation followed by pericarditis and endomyocardial fibrosis (Löffler syndrome).^2^ Cardiac magnetic resonance (CMR) is a powerful diagnostic modality that leads to the early detection of cardiac involvement when endomyocardial fibrosis has not yet occurred and echocardiography results are within normal limits.^3^ A prompt diagnosis of cardiac toxocariasis at a stage where cardiomyopathy is still reversible enables the early initiation of treatment and improvement of cardiac function.

## 2 CASE HISTORY

**FIGURE 1:**
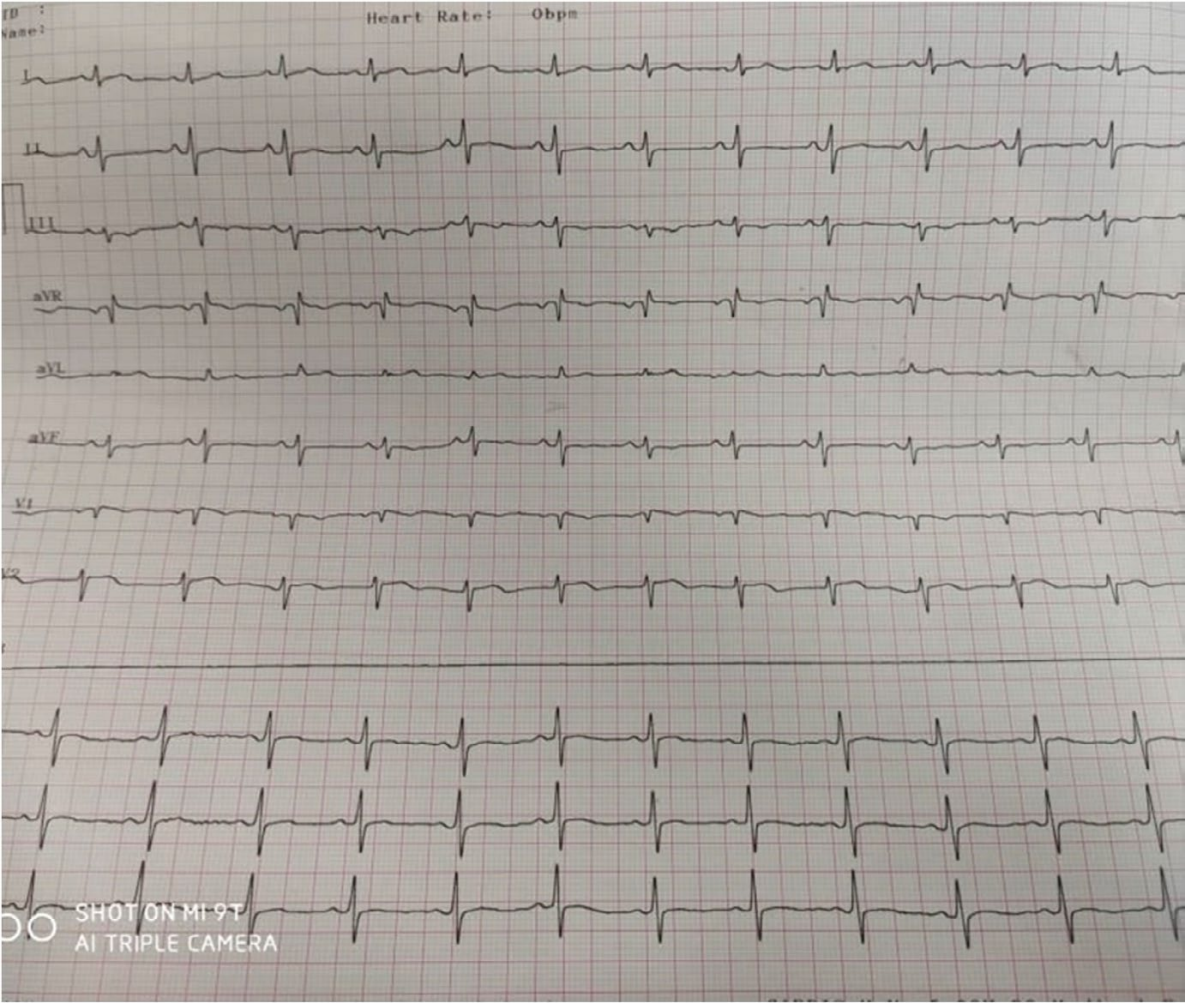
Twelve‐lead ECG in a patient with cardiac toxocariasis demonstrates normal ECG

We describe a 49‐year‐old man with a negative past medical history who presented to another center with ongoing chest pain and dyspnea of 7 days' duration. The patient's familial history was unremarkable. Upon arrival at the previous center, the patient reported a history of prolonged fever, malaise, weakness, abdominal pain, and weight loss of 10 kg within 2 months in the preceding year. A full workup demonstrated an increased erythrocyte sedimentation rate (42 mm/h) associated with significant eosinophilia (the eosinophil count = 12,760/ml [58%]). He also reported close contact with dogs. According to the presence of eosinophilia, a parasitic evaluation was performed, which showed a *Toxocara canis*–positive antibody with 11.8 units of nephelometric turbidity (normal <9 NTU). With the final diagnosis of toxocariasis, he was treated with albendazole (500 mg twice daily) and prednisolone (1 mg/kg/day) for 1 week. After symptom subsidence and a normal complete blood count, he was discharged from that center. In our department, physical examination showed normal findings, including the heart and lung sounds. No abnormality was detected on electrocardiography (Figure 1). Laboratory examination revealed an elevated cardiac troponin level 3 times the normal value, an erythrocyte sedimentation rate of 32 mm/h, and an elevated C‐reactive protein level of 55 mg/L. Transthoracic echocardiography showed mild left ventricular (LV) enlargement with moderate systolic dysfunction and a left ventricular ejection (LVEF) of 35%–40%. LV wall thickness was increased in the apical segments, indicating either mural thrombosis or hypertrabeculation. Additionally, moderate‐to‐severe eccentric mitral regurgitation (effective regurgitant orifice = 0.35 cm^2^) was detected.

## 3 DIFFERENTIAL DIAGNOSIS, INVESTIGATIONS, AND TREATMENT

The differential diagnoses included acute coronary syndrome, myocarditis, cardiac involvement in the setting of a systemic inflammatory condition, and cardiac toxocariasis.

**FIGURE 2:**
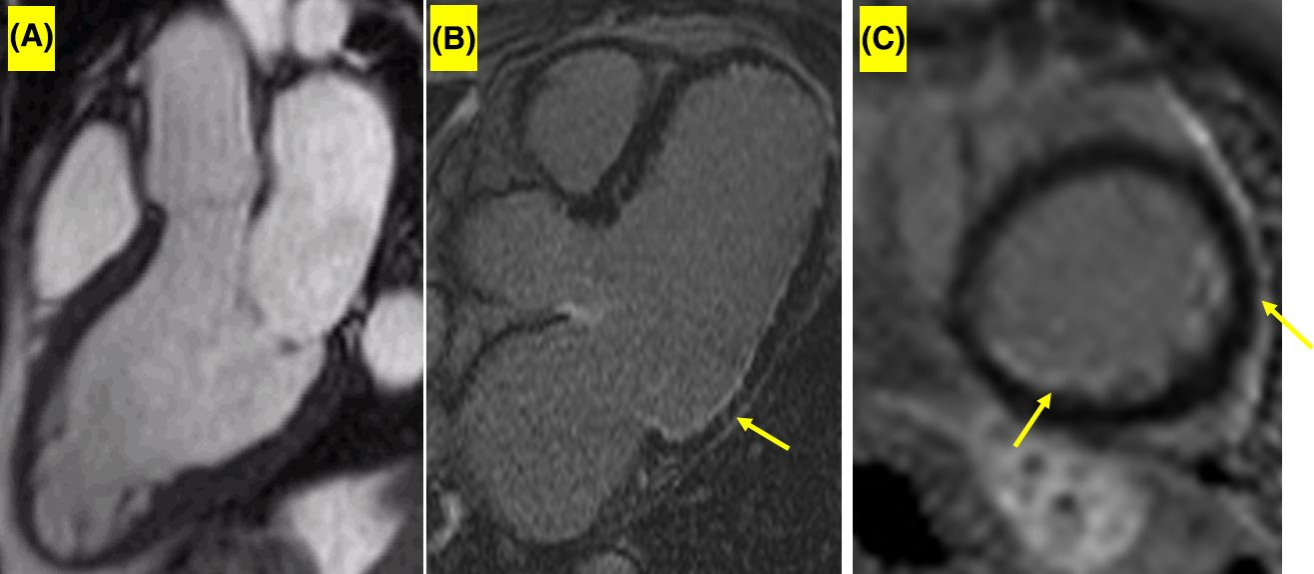
CMR sequence in cardiac toxocariasis. (A) Three‐chamber Cine function CMR, (B) 3‐chamber LGE at the same site of A shows areas of subendocardial fibrosis in mid to apical part of LV inferolateral wall. (C) Short‐axis LGE demonstrates LV lateral and inferior wall subendocardial fibrosis

In the primary center, the patient's clinical and laboratory findings led to coronary angiography (Figure 2), which demonstrated minimal coronary artery disease. The patient experienced two episodes of transient ischemic attacks presenting with hemiparesis and inability to speak. The results of the workups were compatible with the diagnosis of myocardial inflammation. Moreover, the *Toxocara* antibody level was 13 NTU with a mildly increased eosinophil level (1000/µl, 10%).

**FIGURE 3:**
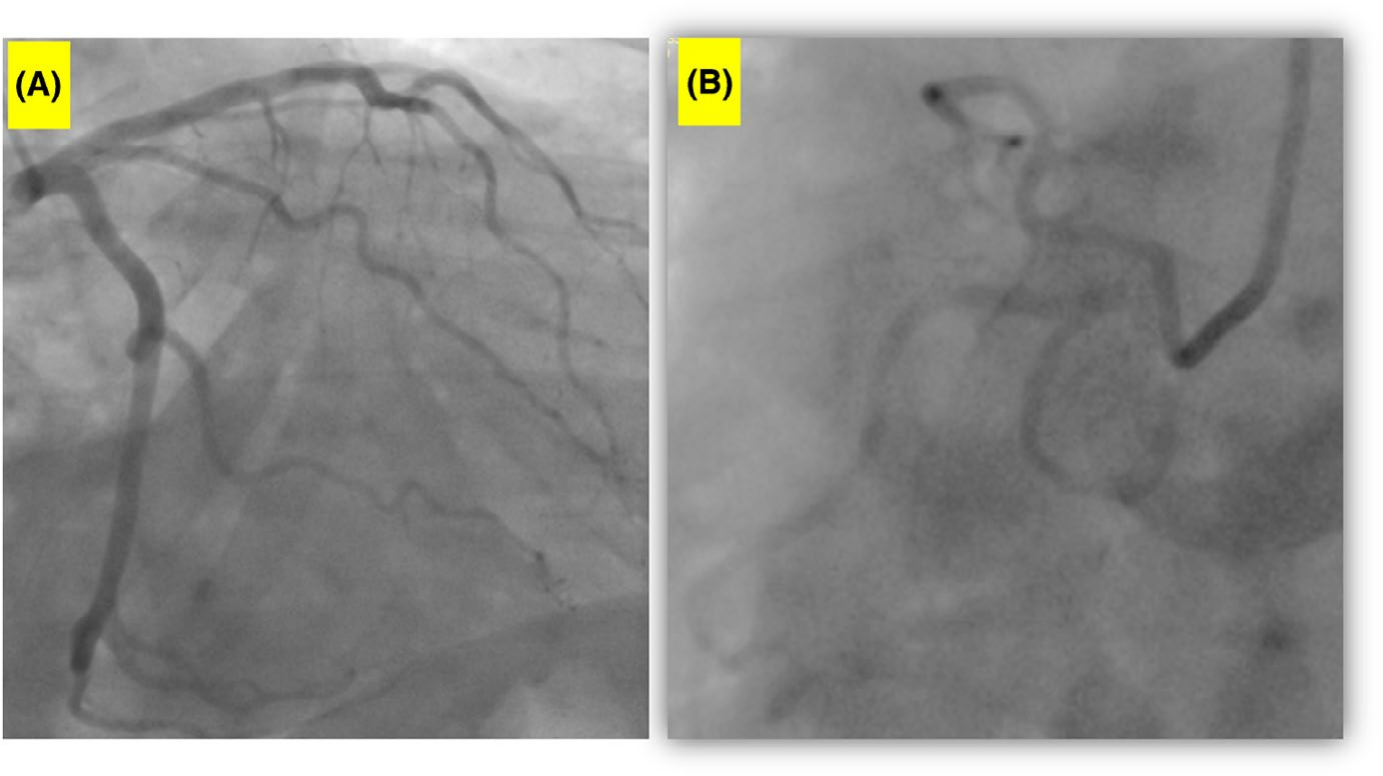
Cardiac angiography shows normal (A) left and (B) right coronary angiography results

The patient was referred to our center for CMR aimed at evaluating cardiac involvement in the context of toxocariasis‐induced HE (Figure 3). On CMR, mild LV enlargement with moderate systolic dysfunction (LVEF = 40%) was detected. There was no evidence of myocardial edema. The late gadolinium enhancement (LGE) sequence revealed extensive subendocardial enhancement in the LV lateral segments, from the base to the apex. No evidence of clot formation was noted in the cardiac chambers. The CMR findings favored endomyocardial involvement in the context of toxocariasis‐induced HE. The diagnosis was further verified by the presence of hepatosplenomegaly. Accordingly, treatment with albendazole (500 mg twice daily) and prednisolone (1 mg/kg/day) was continued for 4 weeks.

## 4 OUTCOME

After 2 weeks of treatment with albendazole and prednisolone, the patient became symptom‐free and the cardiac troponin level returned to the normal range. Consequently, the treatment continued for 2 more weeks on an outpatient basis. An annual control of cardiac function was ordered for the patient.

## 5 DISCUSSION

In this report, we described the CMR findings of a patient with established toxocariasis. CMR demonstrated evidence of HE‐related cardiac involvement.

Toxocariasis is a known and relatively common parasitic disease around the world. It is caused by *Toxocara canis* and *Toxocara cati* with a prevalence of as high as 40% in tropical and subtropical countries.^4^

The clinical findings range from asymptomatic to fatal based on the organs involved. The most common symptoms are prolonged fever, malaise, nausea and vomiting, weight loss, and allergic symptoms.^1^ The predominant laboratory finding is HE, which is often present in infected patients.^2^, ^5^

Humans seem to be accidental hosts, and they are usually infected by soil contaminated with the feces of an infected cat or dog through the gastrointestinal system.^6^ Larvae invade the intestinal wall, enter the circulation, and migrate to the other organs. These events result in an eosinophilic granulomatosis reaction to inhibit further propagation.^2^

Toxocariasis is one of the chief causes of HE, but it is seldom associated with cardiac complications. In a report on 16 children affected with toxocariasis, only one case of cardiac involvement, manifesting itself with myocarditis, was detected.^7^ The most common cardiac manifestation of toxocariasis is myocarditis, followed by pericarditis and endomyocardial fibrosis (Löffler syndrome).^2^

Injury to cardiomyocytes is thought to be the consequence of either direct larval infiltration or immunologic reaction triggered by HE and intense cytokine release.^2^ Endomyocardial injury caused by HE happens in three stages: (1) the necrotizing stage, (2) thrombosis formation, and (3) myocardial fibrosis.^3^ These pathologic events result in restrictive cardiomyopathy.^8^, ^9^ We believe that the CMR manifestations, including a reduced LVEF and a subendocardial LGE pattern, in our patient were related to the fibrotic stage and representative of endomyocardial layer fibrosis.

The advent of CMR has caused a revolution in the diagnosis and management of cardiac diseases, notably in conditions involving the myocardium, in recent decades. While cine steady‐state‐free precession imaging confers potency in functional evaluation, CMR is capable of revealing such tissue characteristics as inflammation and fibrosis via multiple sequences. The short tau inversion recovery sequence has been used for revealing edema for many years. Moreover, the LGE sequence can confer the detection of myocardial fibrosis with acceptable sensitivity. Quantitative mapping techniques improve the sensitivity of CMR tissue characterization significantly. Moreover, further information such as probable clot formation and deformation parameters could be achieved, contributing to a more precise diagnostic workflow.^10^ CMR is a powerful modality to detect cardiac involvement in the early stages where echocardiography is incompetent. It demonstrates either endomyocardial fibrosis or other cardiac manifestations of toxocariasis. The strength of CMR in tissue characterization revealed evidence of definite myocardial involvement in the context of parasitic infection in our patient.^11^

In a systematic review, Kuenzli et al. analyzed reports of cardiac toxocariasis in 24 cases. Cardiac involvement was established by symptoms, electrocardiography, troponin levels, and echocardiography. In 9 cases, an endomyocardial biopsy (EMB) was performed. Three of the samples were positive for parasitic remnants, while the other six biopsies showed nonspecific findings.^5^ Park et al.^12^ described a 57‐year‐old man with worsening dyspnea, HE, and evidence of right ventricular apical fibrosis and thrombosis in CMR. The patient underwent an EMB, which proved inconclusive. Ultimately, a diagnosis of toxocariasis was made with positive serology.

Therefore, although EMB is considered the gold standard for diagnosis, not only do excessive fibrosis and sampling errors render it insensitive^12^ but also the risk of myocardial rupture is unavoidable. However, strikingly, noninvasive modalities such as CMR may guide the diagnosis even in earlier stages.

Our CMR findings illustrated notable subendocardial fibrosis, which we hypothesized to be either due to recurrent infection or due to a sequel of persistent untreated cardiac toxocariasis.

The value of CMR has been proven in cardiomyopathies associated with HE. This imaging modality is highly sensitive and specific in detecting active inflammation, thrombosis, and fibrosis, all of which comprise the characteristic findings of endomyocardial involvement in hypereosinophilic syndrome.^3^, ^9^, ^13^

Cardiac toxocariasis, if not treated swiftly, may lead to restrictive cardiomyopathy, ischemic complications, and embolic events.^2^ An effective course of treatment may, however, result in striking improvement. Therefore, we recommend that CMR be performed with a lower threshold to detect and treat cardiac involvement as early as possible.

## 6 CONCLUSIONS

The timely detection and effective treatment of cardiac complications secondary to HE can help prevent further consequences such as restrictive cardiomyopathy. CMR, with the capability of tissue characterization, is a powerful diagnostic modality for the early diagnosis of cardiac diseases in hypereosinophilic syndrome. Accordingly, we recommend CMR with a lower threshold to recognize and treat possible cardiac involvement as expeditiously as possible.

## CONFLICT OF INTEREST

The authors declare no conflicts of interest.

## AUTHOR CONTRIBUTIONS

NR and SA: contributed to the acquisition of data, the drafting of the manuscript, and the final revision of the manuscript. SAF: contributed to the acquisition of data and the drafting of the manuscript. All 3 authors participated sufficiently in the work.

## ETHICAL APPROVAL

The report was performed in accordance with the ethical standards laid down in the 1964 Declaration of Helsinki and its later amendments or comparable ethical standards. Additionally, it is in accordance with institutional policies vis‐à‐vis human studies.

## CONSENT

Written informed consent was obtained from the patient.

## Data Availability

The datasets generated during the current report are available from the corresponding author on reasonable request.
